# Insight the Luminescence Properties of AlON: Eu, Mg Phosphor under VUV Excitation

**DOI:** 10.3390/ma10070723

**Published:** 2017-06-29

**Authors:** Xian Jian, Hui Wang, Ming-Hsien Lee, Wei Tian, Guo-Zhang Chen, Wen-Qiao Chen, Wei-Wei Ji, Xin Xu, Liang-Jun Yin

**Affiliations:** 1 School of Energy Science and Engineering, University of Electronic Science and Technology of China, 2006 Xiyuan Road, Chengdu 611731, China; jianxian@uestc.edu.cn (X.J.); 15520760583@163.com (H.W.); 15528127896@163.com (W.T.); cwq_uestc@126.com (W.-Q.C.); 2 Department of Physics, Tamkang University, Tamsui, New Taipei 25137, Taiwan; mhslee@mail.tku.edu.tw; 3 School of Physics, University of Sydney, Sydney, NSW 2006, Australia; chengz910624@gmail.com; 4 Laboratory of Materials for Energy Conversion, Department of Materials Science and Engineering, University of Science and Technology of China, Hefei 230026, China; benjee@mail.ustc.edu.cn (W.-W.J.); xuxin@ustc.edu.cn (X.X.)

**Keywords:** AlON, VUV luminescence, first-principle calculation, thermal stability

## Abstract

Owing to high quantum efficiency, adjustable composition and antioxidation properties of oxynitride phosphors, extensive investigations have focused on their photoluminescence properties under low-energy light excitation (UV or blue light). However, the vacuum ultraviolet (VUV) luminescence properties of oxynitride phosphors are rarely researched. Present work studies the structure and VUV luminescence properties of an oxynitride phosphor: AlON: Eu, Mg, which is synthesized by solid-state reaction. Under 147 nm excitation, it was found that AlON: Eu, Mg phosphor shows a blue emission band centered at about 470 nm. The first principle calculation is used to analyze the origin of the VUV absorption. Compared with BaMgAl_10_O_17_: Eu^2+^ phosphor, AlON: Eu, Mg phosphor shows better thermal stability.

## 1. Introduction

Recently, extensive investigations have focused on rare-earth-doped oxynitride phosphors due to their high quantum efficiency, adjustable composition, and antioxidation properties [1,2,3,4]. These characteristics make them successful applications in White-Light Emitting Diode (W-LED). As one kind of oxynitride, γ-aluminum oxynitride with spinel structure (hereafter AlON) is well known as a transparent ceramic in the visible light range and it has superior mechanical properties both at room and high temperature [5], which makes AlON a promising host-lattice for doping transition or rare-earth luminescent ions [6,7,8,9]. In particular, when doped with Eu^2+^ ions, AlON can be developed into an interesting blue-green luminescent material which exhibits a strong emission band centered at 470–500 nm under 310–330 nm light irradiation [10,11,12].

In addition to the general luminescence properties under ultraviolet (UV) or blue light excitation, it has been proved that some of oxynitride phosphors show intriguing luminescence under VUV excitation [13,14]. To the best of our knowledge, there is still no report on the VUV luminescence properties of AlON based phosphors. Therefore, it is spectroscopically interesting to study their VUV luminescence properties.

In this study, Eu doped AlON phosphor with a single spinel structure was prepared by solid-state reaction. Its VUV luminescent properties, as well as the thermal stability, were experimentally determined. First-principle calculations were adopted to propose the possible origin of the absorption in VUV band.

## 2. Results and Discussion

**Figure 1:**
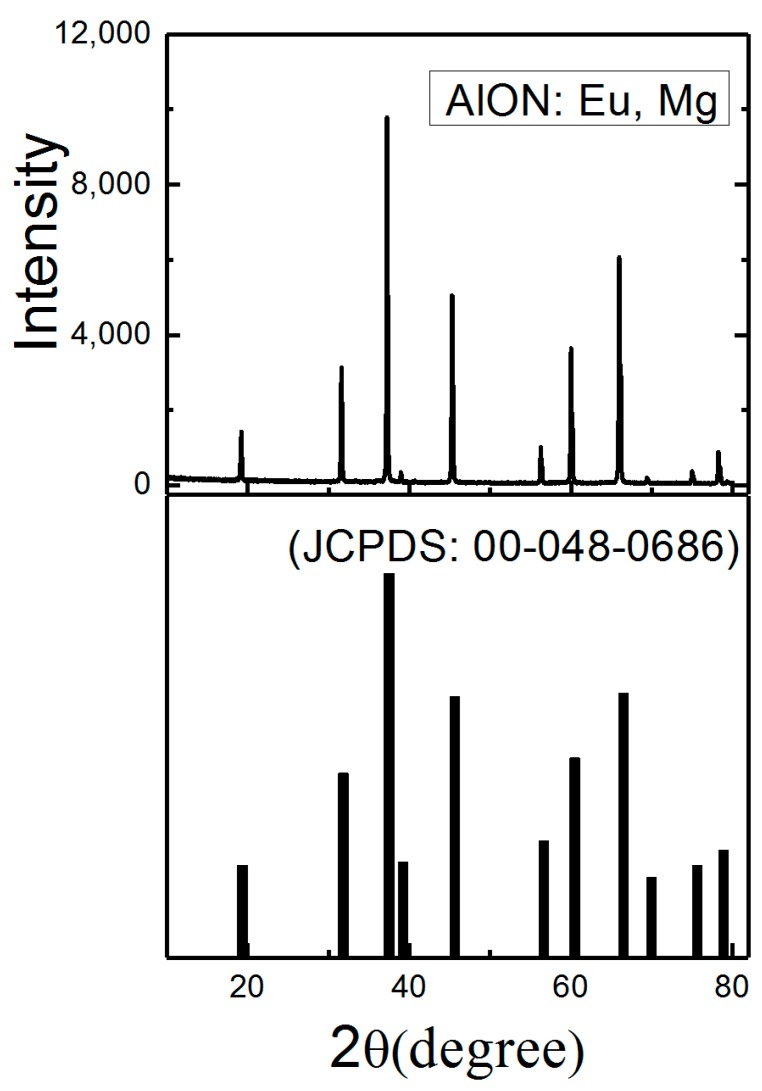
X-ray diffraction (XRD) patterns of AlON: Eu, Mg phosphor.

As reported, the addition of Mg can effectively suppress the impurity of corundum and promote the AlON formation [7]. X-ray diffraction (XRD) patterns of Figure 1 show that the samples have the single AlON phase with spinel crystal structure (JCPDS card No. 00-048-0686, Al_5_O_6_N). The strong XRD peaks of the powders match well with the standard date, indicating the high purity and good crystallinity of the products. Based on XRD peaks, the cell parameter increases from a = 7.95 Å in AlON to a = 8.00 Å in AlON: Eu, Mg due to Mg doping. The measured metallic elemental composition is 0.28 mol% (Eu), 8.91 mol% (Mg) and 90.81 mol% (Al). Therefore, the overall composition of synthesized AlON: Eu, Mg phosphor can be expressed as Eu_0.06_Mg_2.05_Al_20.89_O_29.11_N_2.89_, which is close to the designed composition of Eu_0.046_Mg_2.3_Al_20.654_O_29.346_N_2.654_.

**Figure 2:**
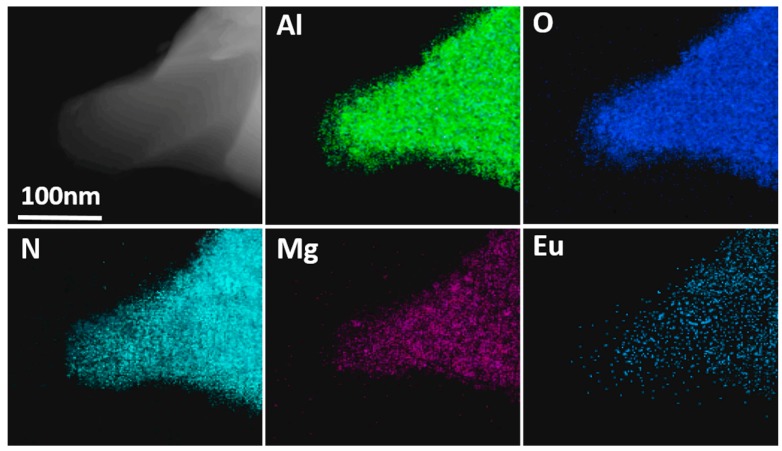
Energy-dispersive X-ray spectrometry (EDS) mapping showing the distribution of Al, O, N, Mg and Eu in AlON: Eu, Mg phosphor.

In order to study element distribution in AlON, energy-dispersive X-ray spectrometry (EDS) mapping analyses are performed in Figure 2. All element mappings are in accordance with the TEM images of the particle shapes. EDS mapping result shows the uniform distribution of Al, O, N and Mg elements in AlON particle, validating the dissolution of Mg into AlON lattice. Although the signal corresponding to Eu is quite weak due to its low concentration, it is still clear to see that the Eu distribution almost matches the particle shape. This indicates that the Eu is dissolved into AlON lattice.

**Figure 3:**
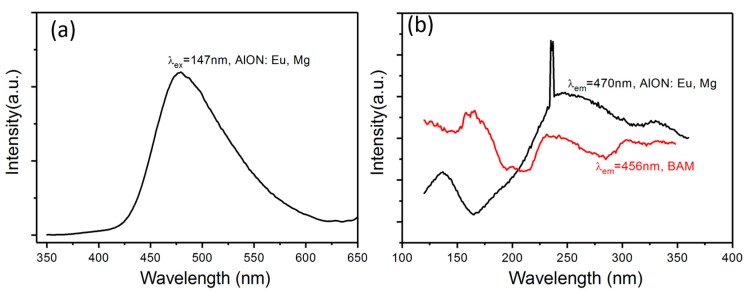
Emission (**a**) and excitation spectra (**b**) of the AlON: Eu, Mg phosphor. The sharp signal in the excitation spectrum at about 240 nm is due to no use of the correct filter, as a consequence of which second order radiation is transmitted.

The previous report has indicated that AlON: Eu, Mg phosphor can be effectively excited by 330 nm UV light and give a strong blue emission [10,12]. Their VUV luminescence property is researched here. Figure 3a exhibits the emission spectrum of the AlON: Eu, Mg phosphor under 147 nm excitation. The emission spectrum is dominated by a blue emission band centered at about 470 nm, which is assigned to the typical 4f^6^5d^1^-4f^7^ transition of the Eu^2+^ ion. Note that the emission spectrum is very broad, meaning that filtering is needed to improve its color purity in actual display applications. No sharp f-f transition lines characteristic for Eu^3+^ are detected, indicating that Eu is mainly present as the divalent valence in AlON: Eu, Mg.

Figure 3b presents the VUV excitation spectrum of the AlON: Eu, Mg phosphor. The spectrum consists of three main broad bands in the VUV to UV range: 120 to 150 nm, 230–270 nm and 300–350 excitation band. According to the previous reports, excitation band from 230 to 270 nm is due to absorption of the AlON host material because the bandgap of AlON is estimated to approximately 5 eV [15,16]. The 300–350 nm excitation band is caused by the absorption of Eu^2+^. Noticeably, there is an obvious absorption from 120 to 150 nm, centered at 136 nm, which is located in the VUV spectrum. It means that AlON: Eu, Mg phosphor can be excited by VUV light and give a blue emission.

Up to now, the exact sites of Eu^2+^ ions in AlON lattice are still unknown since it is a challenging work to make it clear. On account of large difference of ionic radius between Eu^2+^ and Al^3+^, Eu^2+^ ions are expected to locate at some layered structure like EuMgAl_10_O_17_, which has been verified in Eu, Si co-doped AlN [17]. As a comparison, the VUV excitation spectrum of BaMgAl_10_O_17_: Eu^2+^ is included in Figure 3b. There is a significant difference for the 120–270 nm excitation band due to the different host materials. Both phosphors show absorption in the range of 300–350 nm band, which possibly indicates their similar coordination environment around Eu^2+^. To clarify the origin of the first VUV absorption peak, first-principles calculation is performed here. Before that, a clear structure model of AlON: Eu, Mg, Mg is needed. Considering that the VUV absorption is more ascribed to the intrinsic property of AlON host lattice, Eu^2+^ was not included in calculation process because the intrinsic optical property of AlON will not be altered by quite low doping concentration of Eu.

It is generally believed that, for the transparent ceramic γ-AlON, the crystal would have a cubic spinel-type structure, with space group of Fd$\bar{3}$m. Crystallographically, the ideal spinel space group demands totally 24 cations and 32 anions per unit cell (M_24_X_32_ type). In the structure, octahedrons are connected to each other by edge sharing and to the neighbored tetrahedrons by point sharing. To balance charge neutrality in the structure of AlON, the following formula for a constant anion model can be written as [18]:

Al_(64+z)/3_V_(8−z)/3_O_32−z_N_z_,

where V is cation vacancy. It is investigated that γ-AlON is compositionally stable when z = 5, forming Al_23_O_27_N_5_ due to the low concentration of Al vacancy. By comparing the system energy of two structures with different Al vacancy site (Table 1), the model with an Al vacancy in the octahedral sites have the lowest total energy and is therefore more acceptable, which agrees with the previous calculation results based on density functional theory (DFT) [19,20].

**Figure 4:**
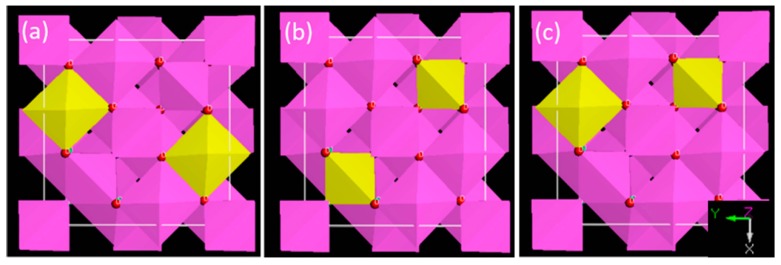
Three Al_21_Mg_2_O_29_N_3_ configurations with different Mg occupation sites.

As expressed in the experimental part, Mg addition is used to suppress the formation of corundum and promise the high purity of obtained AlON powders [7]. Considering approximately two Mg^2+^/Al^3+^ substitutions with unequal charge, O^2−^/N^3−^ substitution is introduced to neutralize the charge, i.e., forming Al_21_Mg_2_O_29_N_3_. To determine the Mg sites in AlON structure, total energy of Al_21_Mg_2_O_29_N_3_ with different Mg occupation sites are calculated, respectively. Totally, three are three possible configurations for Al_21_Mg_2_O_29_N_3_ as shown in Figure 4: (a) both Mg atoms substitute octahedral sites; (b) both Mg atoms substitute tetrahedral sites; (c) one Mg atom substitutes octahedral site and one substitutes tetrahedral site, respectively. Table 2 summarizes the total energy calculated for the different configurations. It is seen that the energy increases in the order:

$$E_{b}\;<\;E_{a}\;<\;E_{c}$$

It stabilizes the AlON crystal that both Mg atoms occupy tetrahedral Al sites in Al_21_Mg_2_O_29_N_3_. In the view of ionic radius (Mg^2+^, 4CN, 0.57 Å), tetrahedral volume matches better with Mg^2+^, which reduces the lattice stress of crystal structure. Therefore, Mg occupation at tetrahedral sites in is more favorable.

**Figure 5:**
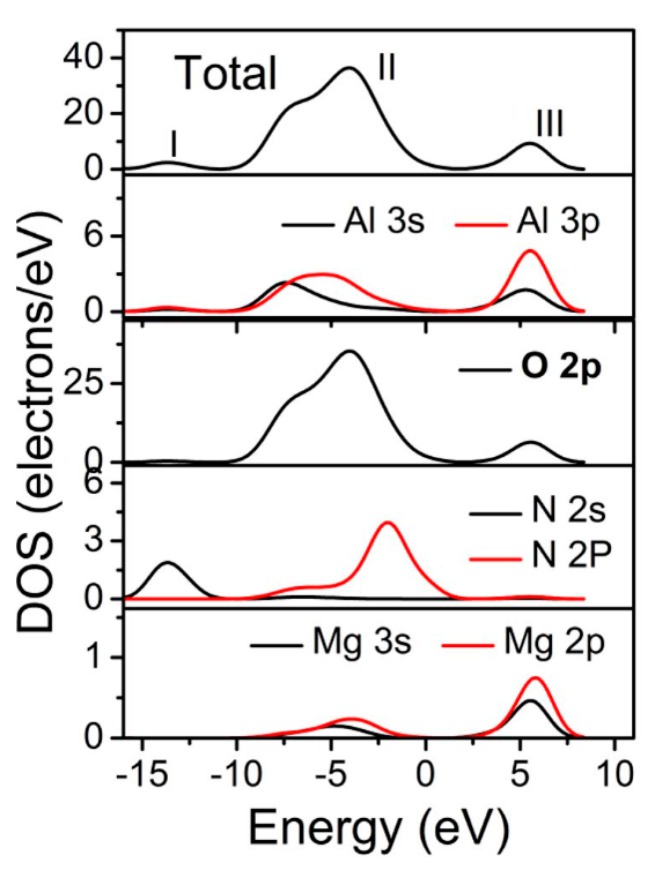
The total and the atom-resolved partial density of states (PDOS) for all the different atoms in Al_21_Mg_2_O_29_N_3_.

Figure 5 shows the total and the atom-resolved partial DOS (PDOS) for all the different atoms in optimized Al_21_Mg_2_O_29_N_3_ configuration. For clarity, the Fermi level is set to zero. Clearly, a sharp peak I mainly comes from N 2s states. The peak II around −4.05 eV is mostly contributed by O 2p state with slight mixings of Al 3s, 3p states. The peak III around 5.3 eV is mainly attributed to Al 3s, 3p, O 2p states. As talked about the VUV excitation spectrum of AlON: Eu, Mg phosphor, the experimental absorption peak around 136 nm corresponds to 9.12 eV. Although the band gap underestimation shown in Figure 5 is typical by DFT (the band gap is smaller than 5 eV), such underestimation does not mean that the higher energy states which results in higher energy (120 nm~160 nm) photon absorption is equally largely underestimated [21,22]. Actually, the energy value of electron transitions just matches the peak of absorption around 136 nm. Thus, it indicates VUV excitation ranging from 120 to 160 nm originates from the O 2p states to Al 3s, 3p states transitions (9.35 eV). This kind of host-lattice excitation associated with Al-O groups in the spinel blocks is also observed in the commercial BaMgAl_10_O_17_: Eu^2+^ and BaAl_12_O_19_: Mn^2+^ phosphor [23,24].

As known, one of luminescence routes is to excite the host lattice firstly, followed by the efficient energy transfer from the excited state of the host lattice to the luminescent ion, resulting in the typical emission of Eu^2+^. The observed luminescence under 147 nm excitation is through this way that an efficient energy transfers from the host lattice to Eu^2+^ ions.

**Figure 6:**
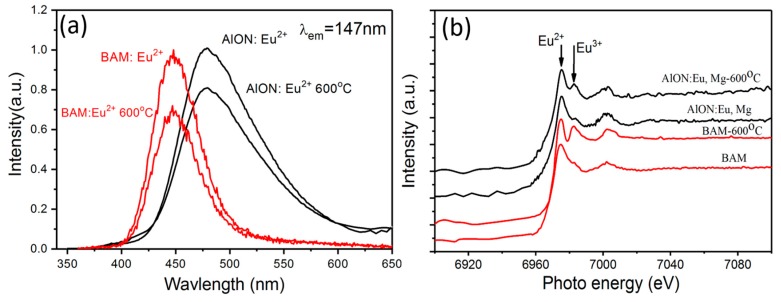
Emission intensity (**a**) and Eu L_3_-edge X-ray absorption near edge structure (XANES) spectra (**b**) of AlON: Eu, Mg and BaMgAl_10_O_17_: Eu^2+^ phosphors before and after annealing at 600 °C for 1 h in air.

Thermal stability is a very important factor for phosphors when working at high temperature. As a popular blue phosphor, BaMgAl_10_O_17_: Eu^2+^ (BAM) with typical layered structure allows intercalation of oxygen ions into its interlayers easily and causes the oxidation of Eu^2+^ into Eu^3+^, leading to severe luminescence degradation [25,26]. On the contrary, AlON consists of a three-dimensional rigid Al (O, N)_4_ network, which will decrease the oxidation degree of Eu^2+^ to Eu^3+^ [27]. As shown in Figure 6, the emission intensity of AlON: Eu, Mg phosphor decreases only 21% after annealing at 600 °C for 1 h in air, while that of the commercial BAM has more than 28% degradation under 147 nm excitation [28]. The improved thermal stability can also be proved by the Eu L_3_-edge X-ray absorption near edge structure (XANES) spectra in the corresponding samples. Two peaks can be clearly seen at about 6977 and 6984 eV, which are due to the divalent and trivalent oxidation states of europium ions, respectively. For AlON: Eu, Mg and BAM, Eu^2+^ is oxidized into Eu^3+^ after heat-treatment at 600 °C in air. However, the oxidation degree differs each other. The relative intensities of the peaks ascribed to Eu^2+^ and Eu^3+^ for the samples suggest that the oxidation degree of Eu^2+^ in AlON: Eu, Mg sample is partly suppressed in the thermal treatment process. The above results indicate AlON: Eu, Mg phosphor shows higher thermal stability than BAM.

## 3. Experimental Section

Materials synthesis. Compared to total cation number, 0.2 mol% Eu and 10 mol% Mg concentration doped AlON (Eu_0.046_Mg_2.3_Al_20.654_O_29.346_N_2.654_) phosphor was synthesized by conventional solid-state reaction. Mg element was introduced to promote the high purity of AlON. The powder mixtures of Al_2_O_3_ (Sinopharm Chemical Reagent Co. Ltd., Shanghai, China), AlN (Sinopharm Chemical Reagent Co. Ltd., Shanghai, China), MgO (Sinopharm Chemical Reagent Co. Ltd., Shanghai, China), and Eu_2_O_3_ (Sinopharm Chemical Reagent Co. Ltd., Shanghai, China) were well mixed in a Si_3_N_4_ mortar by grinding. The resulting mixtures were fired in BN crucibles under N_2_ atmosphere for 2 h at 1800 °C. The samples were heated at a constant rate of 360 °C/h and cooled to room temperature naturally.

### 3.1. Material Characterization

The phase formation was analyzed by an X-ray diffractometer (Model PW 1700, Philips Research Laboratories, Eindhoven, The Netherlands) using Cu K_α_ radiation at a scanning rate of 0.5 degree/min. The composition analysis was performed by transmission electron microscopy-energy dispersion X-ray spectroscopy (Model 2100F, JEOL, Tokyo, Japan). Metallic elements content was analyzed by inductively coupled plasma atomic emission spectrometer (ICP-AES) (Optima 7300DV, Perkin Elmer Corporation, Waltham, MA, USA). The VUV excitation and emission spectra were measured at the VUV spectroscopic experimental station on beam line U10B of the National Synchrotron Radiation Laboratory of China. The synchrotron radiation was monochromatized through a Seya-Namioka monochromator and the signal was received by a Hamamatsu R456 photomultiplier. The X-ray absorption spectra at the Eu L3-edge of AlON: Eu, Mg were measured at the beamline of BL14W1 at Shanghai Synchrotron Radiation Facility.

### 3.2. Computational Methodology

The theoretical calculations were performed using the Cambridge Sequential Total Energy Package (CASTEP) code [29,30]. A plane wave basis set with kinetic energy cutoff at 500 eV was employed, with the electron-ion interaction accounted for through the use of ultrasoft pseudopotentials [31]. Such E_cut allows the convergence of total energy to be within 0.1 eV/atom. The Perdew-Burke-Enzerhof form [32,33] of the generalized gradient approximation (GGA) was used to describe the exchange-correlation interactions. Actual spacing of *k*-point sampling is 0.067 Ang^−1^. Monkhorst-Pack sets of *k* points = 1 × 1 × 1 was sufficient, and we set SCF tolerance threshold to be 1.0 × 10^−5^ eV/atom. In Mg doped AlON cell structure, two Al atoms are substituted by same quantity of Mg atoms. Then the cell-varying geometry optimization is processed for relaxing crystal lattice, giving the supercell parameters of a = 8.08 Å, b = 8.05 Å and c = 8.11 Å. The yielded lattice parameters errors (when compared with experimental values: 8.00 Å) Err (a, b, c) = (1.00%, 0.63%, 1.38%), respectively.

## 4. Conclusions

AlON: Eu, Mg phosphor is synthesized by solid-state reaction. Its structure and VUV luminescence properties are investigated. The experimental results indicate that Al, O, N, Mg and Eu elements are successfully dissolved into AlON lattice. Under 147 nm excitation, the phosphor shows a blue emission band centered at about 470 nm. First principle calculation results indicate that the VUV excitation ranging from 120 to 160 nm originates from host-lattice excitation of Al-O groups in the spinel blocks. Compared with BaMgAl_10_O_17_: Eu^2+^ phosphor, AlON: Eu, Mg phosphor shows higher thermal stability due to the three-dimensional rigid Al (O, N)_4_ network.

## Figures and Tables

**Table 1:** Total energy E (eV) and relative energies ΔE (eV) of Al_23_O_27_N_5_ with Al vacancy at different sites obtained from the generalized gradient approximation (GGA) calculations.

Number	Al Vacancy	E (eV)	ΔE (eV)
1	octahedral site	−14,534.34	0
2	tetrahedral site	−14,521.69	12.65

**Table 2:** Total energy E (eV) and relative energies ΔE (eV) of Al_21_Mg_2_O_29_N_3_ with Mg at different sites obtained from the GGA calculations.

Number	Mg Sites	E (eV)	ΔE (eV)
a	octahedral sites	−16,689.96	13.04
b	tetrahedral sites	−16,703.00	0
c	octahedral and tetrahedral sites	−16,687.07	15.93
